# Water, sanitation, and intimate partner violence: Insights from Kibra Slums, Nairobi

**DOI:** 10.7189/jogh.14.04141

**Published:** 2024-06-28

**Authors:** Stephen Ombija, Hesborn Wao, Tammary Esho

**Affiliations:** 1 Amref International University (AMIU), Nairobi, Kenya; 2 African Population and Health Research Centre (APHRC), Nairobi, Kenya

## Abstract

**Background:**

Intimate partner violence (IPV) poses significant health and social challenges for women, particularly in slums characterised by limited access to basic amenities like water and sanitation facilities. This study aimed to investigate the association between accessibility of water, sanitation and hygiene (WASH) facilities and IPV among women in Kibra, Nairobi county, Kenya.

**Methods:**

A cross-sectional study design utilising a modified Demographic Health and Survey questionnaire was conducted among women aged 15–49 in Kibra slums. Data on water and sanitation accessibility and IPV experiences were collected from 1068 participants. Quantitative analysis by use of logistic regression, was conducted to assess associations between WASH accessibility and IPV.

**Results:**

Among the participants, 64.0% reported experiences of IPV. Women who had access to water inside household; adjusted odds ratio (AOR) = 0.44 (95% CI = 0.31–0.64) and sanitation AOR = 0.57 (95% CI = 0.37–0.88) had decreased odds of experiencing IPV whereas reliance on external water sources such as outside pipes AOR = 18.18 (95% CI = 8.62–38.33) or vendors AOR = 14.42 (95% CI = 6.88–30.24) had heightened IPV vulnerability.

**Conclusions:**

Access to clean water and sanitation is associated with reduced likelihood of women experiencing IPV in slums whereas access to water outside household is associated with increased likelihood of experiencing IPV. Connecting households with water to improve access and construction of adequate sanitation facilities may protect women against intimate partner violence in slums.

Intimate partner violence (IPV) is a pervasive public health problem that involves violation of human rights [1,2]. It is influenced by intricate sociocultural, economic, and environmental determinants [3]. Different studies have identified IPV as a contributor of poor health outcomes among women [4]. It has been linked to deprived psychological and physical health [5] and homicides [6]. Between 2000 and 2018, IPV was estimated to have affected 27% of women aged from 15–49 years globally [2]. Despite efforts by countries to address sustainable development goals of eliminating violence against women (SDG 2.5), solutions not only need to focus on identifying economic and behavioural risk factors but also on understanding environmental factors [1].

The unequal access to water and adequate sanitation facilities [1], particularly among marginalised communities such as slums, disproportionately impacts women, intensifying their vulnerabilities to IPV. Factors such as long distances to water collection points [1], heightened exposure to harassment or assault, and the lack of proper sanitation facilities intertwine with economic constraints [7], cultural norms, and roles [8], amplify stressors within households resulting to IPV as indicated by Sabri and Campbell [9].

Previous studies have indicated water insecurities in households elevate IPV exposure as punishment for failure to perform social household duties such as cleaning and cooking [8]. In India, a study indicated that nearly a quarter of women in slums have ever experienced IPV [10] with factors ranging from lack of sanitation facilities, Sabri and Campbell [9] early marriage, alcoholism, employment and women’s justification of wife-beating [10]. Similarly, a study conducted in the slums of Nairobi in 2000 found that 16% of women had experienced IPV [11], rates that continue to rise [12].

The health and well-being of the people of marginalised urban settlements such as the Kibra slums is greatly affected by the convergence of socioenvironmental factors. One of the significant but as-yet-unexplored factors is the complex web of relationships between IPV and the availability of basic services such as water and sanitation. In the difficult socio-environmental context of Kibra slums, this research paper investigates the empirical investigation of the association between IPV and the access of water and sanitation infrastructure

## METHODS

A cross-sectional study design was implemented utilising a modified household survey derived from the Demographic and Health Survey (DHS) [13]. In this study, seven out of the twelve informal villages in Kibra slums were randomly chosen through a lottery method. The study focused on women aged 15–49 residing in the slum areas of Kibra sub-county, Nairobi, Kenya, who had lived there for a minimum of six months and gave informed consent. The study excluded women who had experienced violence from someone other than their intimate partner or encountered lifetime IPV.

The modified DHS module employed in our study effectively gathered data on several key variables including access to water within the past 24 hours, primary water source, and access to sanitation facilities within the past 24 hours. Sociodemographic and behavioural factors such as age, income, employment, parity, alcohol intake, and childhood violence exposure were gathered and included in the model as controls. Additionally, we collected information on IPV, the primary outcome variable of interest. Using Epi info version 7.2.5.0, a larger sample size (n = 1068) than the calculated sample size (n = 384) was interviewed using systematic random sampling by the selection of every 10th household.

### Dependant and independent variables

The outcome variable in this study was past year experiences of IPV as per the DHS survey [13]. To measure IPV, we utilised the assessment tool developed by the World Health Organization (WHO), which is a comprehensive instrument covering three dimensions of IPV: physical (consisting of six items), emotional (three items), and sexual (three items) [13,14]. Past year IPV was defined as the proportion of ever partnered women who have experienced one or more acts of IPV, Okedare and Fawole [15]. Analysis of IPV scores depended on the women's reports of IPV within the past twelve months. Physical violence involves a partner being attacked by a knife or object/threatened with a knife or object, hit/slapped/punched, pushed/kicked/shaken, or choked/strangled [13,14]. Emotional violence focused on the subscale of three questions. The respondent was asked if the partner has ever insulted/ humiliated her in front of others, threatened to hurt her/threatened to hurt someone she cares about and insult /make the respondent feel bad about herself [13,14].The study participant was asked if the partner has ever physically forced her to have sexual intercourse, physically forced her to perform any other sexual acts she did not want to and if forced by threats in any other way to perform sexual acts she did not want to [13,14]. Each response was quantified on a dichotomous scale of (1 = Yes, 0 = No).

The primary interest in this study was to assess the association between the accessibility of WASH and IPV. Women were asked about their primary source of drinking water and if they had access to water and sanitation facilities within their households. Ready access to water was defined as having access to water without effort or time needed to fetch it and make it safe [16]. Source of primary water for use could be inside household taps- outside tap/Public Tap/Well- or from vendors/tanker/burst pipe/streams. These responses were scored on a scale of 0 for owning inside household tap- 1 for accessing water through outside tap/Public tap/Well- and 2 for accessing water through vendors/tanker/burst pipes/streams. A binary response was quantified on a dichotomous scale of (1 = Yes, 0 = No) of the response given by the women on accessing water and sanitation facilities within the last 24 hours.

### Covariates

Sociodemographic and behavioural factors such as age, income, employment, parity, alcohol intake, violence acceptance, childhood violence exposure, and other variables like education level were included in the model due to their demonstrated associations with IPV [10,14,17]. The data are presented as shown in Table S6 in the **Online Supplementary Document**.

#### Statistical analyses

Multivariable logistic regression (MLR) was used to estimate the association between accessibility of WASH facilities and IPV. In our regression models, we were able to analyse independent variables into crude and adjusted models. By dropping non-significant variables during modelling, we were able to control for confounding effect. Multivariable logistic regression (MLR) was used to estimate the association between accessibility of WASH facilities and IPV.

## RESULTS

**Figure 1:**
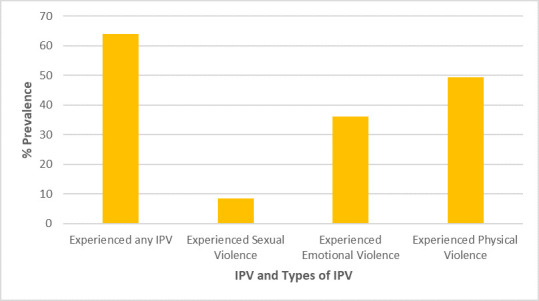
Prevalence of IPV and its sub-forms. IPV – intimate partner violence

**Figure 2:**
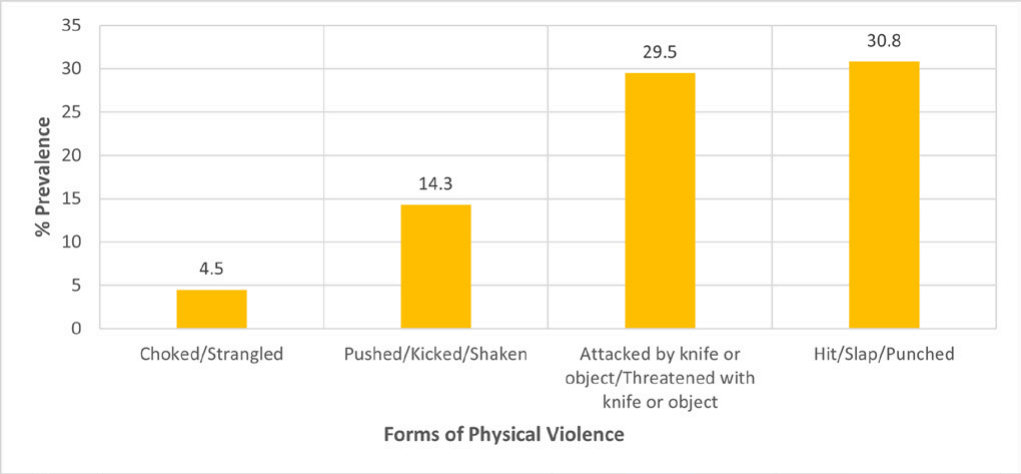
Prevalence of the different types of physical violence.

**Figure 3:**
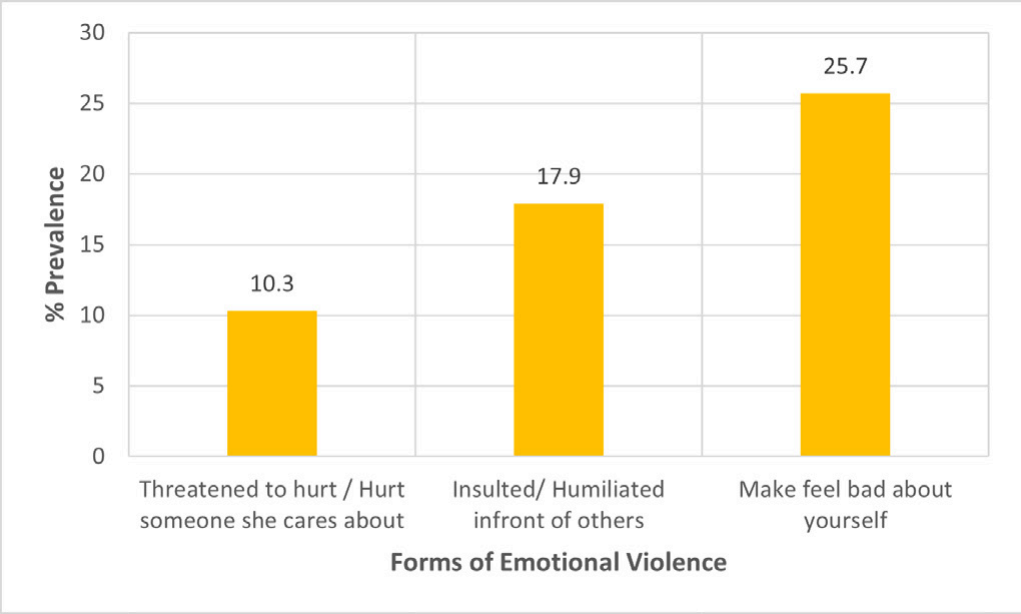
Prevalence of the different types of emotional violence.

**Figure 4:**
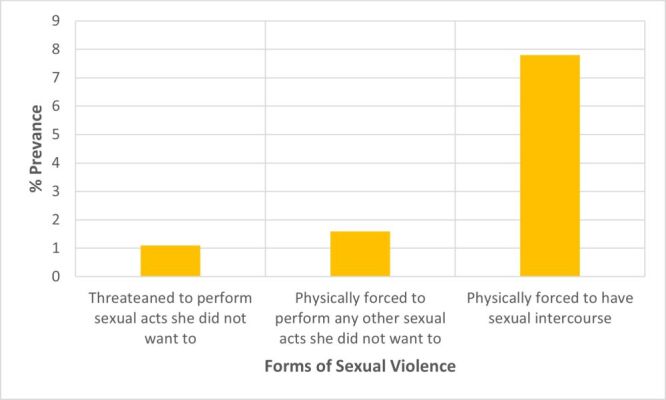
Prevalence of the different types of sexual violence.

A total of 684 out of 1068 women (64%) experienced some form of intimate partner violence (IPV) (**Figure 1**). In this group, 90 (8.4%), 385 (36.1%), and 528 (49.4%) women reported occurrences of sexual, emotional, and physical violence, respectively. The predominant physical violence was being hit/slapped/punched, accounting for 30.8% (**Figure 2**). Women who reported cases of choke/strangled were in the minority at 4.5% in this category. Making one feel bad about themselves (25.7%) was the most common form of emotional violence out of the three possible subcategories of emotional violence (**Figure 3**). Women who reported having been physically forced to have sexual intercourse (7.8%) was the most common form of sexual violence as shown in **Figure 4**.

The study also revealed distinct IPV prevalence across various demographic and relational factors among women in Kibra sub-county (**Table 1**). Notably, the age group of 35 years and above exhibited the highest overall prevalence of IPV, reaching 67.3%. This prevalence peaked at 70.6% when the partner or ex-partner fell within the same age bracket. Furthermore, a notable observation was the heightened prevalence of IPV among women with an age disparity of 10 years or more, where the prevalence surged to 78.1% (**Table 1**).

**Table 1:** Demographics of women and IPV rates

		Physical violence	Emotional violence		Sexual violence	IPV
**Variable**	**n (%)**		**n (%)**	**n (%)**		**n (%)**
Participant's age in years						
*15–24*	111 (42.2)		82 (31.2)	20 (7.6)		149 (56.7)
*25–34*	256 (51.6)		176 (35.5)	44 (8.9)		327 (65.9)
*35 and above*	161 (52.1)		127 (41.1)	26 (8.4)		208 (67.3)
Partner's age in years						
*15–24*	37 (39.0)		36 (37.9)	10 (10.5)		55 (57.9)
*25–34*	188 (43.2)		132 (30.3)	37 (8.5)		249 (57.2)
*35 and above*	303 (56.3)		217 (40.3)	43 (8.0)		380 (70.6)
Age difference						
*0–2*	109 (41.8)		86 (33.0)	22 (8.4)		153 (58.6)
*3–5*	197 (46.8)		149 (35.4)	35 (8.3)		257 (61.1)
*6–10*	168 (55.3)		118 (38.8)	26 (8.6)		210 (69.1)
*Above 10*	54 (65.9)		32 (39.0)	7 (8.5)		64 (78.1)
Participant’s education level						
*Primary*	262 (60.8)		184 (42.7)	53 (12.3)		312 (72.4)
*Secondary*	217 (42.8)		171 (33.7)	30 (5.9)		307 (60.6)
*College/TVET*	40 (37.0)		24 (22.2)	7 (6.5)		51 (47.2)
*University*	9 (40.9)		6 (27.3)	0 (0.0)		14 (63.6)
Partner’s education level						
*Primary*	164 (54.5)		122 (40.5)	30 (10.0)		211 (70.1)
*Secondary*	275 (49.6)		208 (37.6)	44 (7.9)		359 (64.8)
*College/TVET*	71 (43.3)		42 (25.6)	14 (8.5)		88 (53.7)
*University*	18 (36.7)		13 (26.5)	2 (4.1)		26 (53.1)
Marital status						
*Single*	44 (38.6)		38 (33.3)	8 (7.0)		67 (58.8.2)
*Married*	371 (50.6)		258 (35.2)	53 (7.2)		478 (65.2)
*Divorced*	51 (60.0)		39 (45.9)	15 (17.7)		59 (69.4)
*Cohabiting*	58 (47.2)		49 (39.8)	14 (11.4)		76 (61.8)
*Widowed*	4 (30.8)		1 (7.7)	0 (0.0)		4 (30.8)
Partner has another wife						
*Yes*	111(62.4)		90(50.6)	29(16.3)		140 (68.7)
*No*	411(46.6)		294(33.3)	61(6.9)		538 (60.9)
Number of children						
*0–1*	130(40.4)		96(29.8)	26(8.1)		182 (56.5)
*2–4*	338(52.9)		236(36.9)	54(8.5)		423 (66.2)
*5 and above*	60(56.1)		53(49.5)	10(9.4)		79 (73.8)
Participant's employment status						
*Employed*	68 (54.0)		40 (31.8)	15 (11.9)		81 (64.3)
*Self-employed*	166 (52.4)		124 (39.1)	23 (7.3)		214 (67.5)
*Unemployed*	294 (47.0)		221 (35.4)	52 (8.3)		389 (62.2)
Partner's employment status						
*Employed*	248 (48.3)		175 (34.1)	45 (8.8)		315 (61.4)
*Self-employed*	158 (51.5)		113 (36.8)	22 (7.2)		208 (67.8)
*Unemployed*	122 (49.2)		97 (39.1)	23 (9.3)		161 (64.9)
Average household income						
*Less than 15 000*	389 (50.2)		284 (36.7)	70 (9.0)		499 (64.4)
*15 000 and above*	139 (47.4)		101 (34.5)	20 (6.8)		185 (63.1)

IPV – intimate partner violence, TVET – technical and vocational education and training

Women with only a primary level of education also showed a high prevalence rate of 72.4%. Similarly, IPV prevalence was notably higher when the partner or ex-partner had received primary education, reaching 70.1% as shown in **Table 1**. Divorced women also exhibited a substantial prevalence of 69.4%, shedding light on the vulnerability within this demographic (**Table 1**). The category with the highest overall IPV prevalence was those with five and above children (73.8%), with the least 0 to 1 child at 56.5% (**Table 1**).

An analysis of IPV concerning access to water, sanitation and different water sources revealed different rates as shown in **Table 2**. Notably, women lacking access to water reported higher rates of physical violence (60%), emotional violence (35.6%), sexual violence (10.5%), and an overall IPV prevalence of 71.6% (**Table 2**). In contrast, among those with access to water, the overall IPV prevalence was 56.5%, with corresponding rates for physical violence at 38.8%, emotional violence at 35.5%, and sexual violence at 6.4% (**Table 2**).

**Table 2:** Demographics of women and IPV rates

	Physical violence		Emotional violence		Sexual violence	IPV
**Variable**		**n (%)**	**n (%)**	**n (%)**		**n (%)**
Access to primary water						
*No*		321 (60.0)	196 (36.6)	56(10.5)		383 (71.6)
*Yes*		207 (38.8)	189 (35.5)	34(6.4)		301 (56.5)
How primary water source is accessed						
*Inside Households*		3 (75.0)	0 (0.0)	0 (0.0)		3 (75.0)
*Outside tap/public tap/well*		235 (57.7)	160 (39.3)	42 (10.3)		287 (70.5)
*Vendor/tanker/burst pipe/stream*		290 (44.1)	225 (34.2)	48 (7.3)		394 (60.0)
Access to toilet						
*No*		221 (62.4)	128 (36.2)	39 (11.0)		255 (72.0)
*Yes*		307 (43.0)	257 (36.0)	51 (7.1)		429 (60.1)

IPV – intimate partner violence

Further analysis of IPV prevalence based on different water sources accessed shed light on nuanced variations. Women with access to water inside households exhibited a notably high overall IPV rate of 75% as shown in **Table 2**. Similarly, those accessing water from outside taps, public taps, or wells experienced an overall IPV rate of 70.5%, while those relying on vendors, tankers, burst pipes, or streams reported a rate of 60.0%. Women who lacked access to toilets demonstrated higher IPV prevalence (72%) compared to women who had access to toilets (**Table 2**).

### Water, sanitation and IPV

As shown in **Table 3**, our bivariate and multivariate analysis revealed substantial associations between access to water, different water sources, sanitation facilities, and the likelihood of experiencing IPV. Specifically, women with access to water in the past 24 hours demonstrated a significantly reduced likelihood of experiencing IPV compared to women who lacked access crude odds ratio (COR) = 0.51 (95% CI = 0.50–0.66). Furthermore, women relying on outside taps, public taps, or wells exhibited a notably higher likelihood of experiencing IPV compared to those accessing water inside households COR = 2.75 (95% CI = 1.68–4.50). Similarly, women obtaining water from vendors, tankers, burst pipes, or streams faced higher likelihood of IPV COR = 3.88 (95% CI = 2.33–6.45) compared to women who had access to water inside households. Additionally, our bivariate analysis highlighted the significance of sanitation facilities, demonstrating that women with access to toilets in the past 24 hours had a significantly diminished likelihood of experiencing IPV in contrast to those without access COR = 0.58 (95% CI = 0.44–0.77). Modelling for adjusted odds ratio (AOR) demonstrated a notable persistence of WASH accessibility and IPV, consistent with the findings of the univariate analysis. Women who had access to water AOR = 0.44 (95% CI = 0.31–0.64) or toilet AOR = 0.57 (95% CI = 0.37–0.88) in the past 24 hours had decreased odds of experiencing IPV. Women who accessed primary water from outside taps/public taps/well AOR = 18.18 (95% CI = 8.62–38.33,) or from vendors/tanker/burst pipe/stream AOR = 14.42 (95% CI = 6.88–30.24) had increased odds of experiencing IPV.

**Table 3:** Binary and multivariate logistic regression of water, access to water and sanitation, and IPV

Variable	COR (95% CI)	*P*-value	AOR (95% CI)	*P*-value
Access to primary water				
No* (Reference)	-	-	-	-
*Yes*	0.51 (0.50–0.66)	<0.001	0.44 (0.31–0.64)	<0.001
How primary water source is accessed				
Inside households* (Reference)	-	-	-	-
*Outside tap/public tap/well*	2.75 (1.68–4.50)	<0.001		<0.001
*Vendor/tanker/burst pipe/stream*	3.88 (2.33–6.45)	<0.001	14.42 (6.88–30.24)	<0.001
Access to toilet				
No* (Reference)	-	-	-	-
*Yes*	0.58 (0.44–0.77)	<0.001	0.57 (0.37–0.88)	0.01

AOR – adjusted odds ratio, CI – confidence interval, COR – crude odds ratio

*Reference group during regression analysis.

Controlled covariates such as level of education, age, parity, childhood violence exposure, were associated with IPV and are provided in the supplementary materials. These findings underscore the significant impact of water, specific water sources and access to sanitation facilities on the likelihood of experiencing IPV among women in slums. The stark contrast in odds ratios emphasises the critical role of household-based water access and sanitation facilities in providing a protective environment against IPV, highlighting the urgent need for improved WASH infrastructure in marginalised communities to safeguard women's well-being.

## DISCUSSION

In this study, we examined different factors associated with IPV with bias geared towards accessibility of WASH in slums. We found that 71.6% of women who had no access to water and 72% of women who had no access to a toilet reported a form of IPV. These findings align with a multi-country survey in SSA on IPV as reported by Guli and Geda [18] that has consistently highlighted the high prevalence of IPV in slums associated with lack of water and sanitation facilities. However, to achieve the objectives of this study, we controlled for other covariates to analyse the association between WASH accessibility and IPV exposure.

Women who had access to water in the past 24 hours had reduced odds of IPV (AOR = 0.44; 95% CI = 0.31–0.64) compared to their counterparts. Various studies have observed that enhanced access to water facilities in resource-constrained environments such as slums, has the potential to positively impact relationships. For instance, in Vanuatu [19], a reported case where a man ceased physically harming his wife after she no longer needed to seek his assistance in fetching water after an improved water source. Other positive effects of water initiatives reported were increased respect and support for women by men [19], reduced conflict between husbands and wives as observed in Mozambique [20], changed division of labour, and increased ability of women to negotiate with husbands [19]. Notably, increased household income has been associated with reduced incidence of IPV in multiple studies. In Kenya, [21] a study on increased household access to water through piped water, reduced the work of women and girls hence facilitated home garden and livestock production resulting to increased household incomes [21]. Moreover, an intervention on improved access to water in rural areas in Kitui Kenya, reported an increase in improved relationships, Zolnikov and Salafia [22]. In contrast to what was reported earlier, households could experience difficulties in irregular meal times, infrequent family conversations and irritation due to lack of hygiene as reported by Zolbokiv and Salafia [22].

We found that women who had access to water sources from outside the household, had increased odds of IPV compared to their counterparts. These results are similar to a study conducted in Nepal that examined the relationship between IPV exposure and sub optimal access to water [8]. This association can be related to the fact that household water management in many households is considered a woman’s responsibility [8] hence household water insecurity could increase women’s exposure to IPV. Water access from outside households is not always sustainable and promising in slums since it depends on vendors and competition exists among other slum dwellers [1]. This might result in an increase in round time for fetching water increasing the partner’s insecurities hence exacerbating IPV [1]. As reported in a study in SSA on relationship between round up time in accessing water and IPV [1], it was found that women who took 30 minutes to access water away from households had increased odds of severe and less severe IPV [1]. This has been associated with mistrust of partners of the women fetching water, which happens when wives take longer than expected to fetch water [23]. Limited access to water can lead to economic hardship and stress within households, which in turn can escalate conflict and contribute to IPV. Economic stressors, compounded by inadequate water infrastructure and sanitation facilities in slums settings, may heighten tensions between intimate partners and increase the likelihood of violence as a response to financial strain or perceived resource scarcity [1].

Women who had access to sanitation facilities in the past 24 hours had lower risk of IPV AOR = 0.57 (95% CI = 0.37–0.88) in contrast to their counterparts. A study on sanitation interventions in Vietnam revealed positive changes in communication between men and women in households, and increased self-confidence among the women [24]. These improvements not only altered the nature of domestic chores related to water use in sanitation facilities but also, in some cases, prompted men to share in these responsibilities. This redistribution of roles resulted in a more equitable distribution of household responsibilities between women and men hence limiting IPV [24]. Research conducted in urban slums in India and South Africa where sanitation facilities were located far from people’s homes showed women were more likely to experience IPV [25]. Women were likely to be scolded for taking longer when attending to sanitation facilities outside homes [25].

Covariates associated with IPV included respondent's primary education level, village of residence, exposure to intergenerational violence transmission (IGT) for both the woman and the partner, an alcoholic partner, and seeking help. Women with primary education were approximately twice likely to experience IPV compared to those with tertiary education, consistent with existing literature indicating lower IPV odds among highly educated women [26]. Similarly, women residing in Kambi Muru were prone to IPV compared to those from Gatwekera. Conversely, women from Lindi, Laini Saba, and Mashimoni villages showed lower odds of IPV exposure. These findings resonate with previous research indicating regional variations in IPV acceptance [27]. Additionally, this study observed an association between IPV and IGT [7], particularly among women and those with partners exposed to IGT. Having an alcoholic partner increased the risk of IPV, consistent with prior research linking alcohol use to IPV [14]. Likewise, women who sought help were likely to experience IPV in this study as detailed in an Indian slum research on IPV disclosure [28]. Information on covariates and IPV has been provided in the supplementary materials.

To effectively prevent IPV, it's imperative to address its underlying causes. This study stresses some of the background factors by unveiling the intricate relationship between water, sanitation and IPV within Kibra slums. Findings suggest that IPV stems from multifaceted factors intertwined with the environment and the social context. By integrating strategic WASH infrastructure into urban planning, universal access to water and sanitation particularly in slum areas may be achieved. This approach is essential in mitigating tensions arising from women having to seek these resources outside their households. Additionally, fostering awareness to challenge societal norms against IPV is crucial. Implementing advocacy and communication efforts against IPV as part of public health initiatives, ideally integrated into the educational curricula, holds a promise in breaking the cycle of violence, as childhood exposure to IPV can contribute to future perpetration and being victims.

### Limitations

While this study provides insights and literature into association between accessibility of WASH and IPV, it has some limitations. The study design was cross-sectional and quantitative. Qualitative analysis was not included which could provide rich insights into behaviour outcomes. With cross-sectional study design, we could not make causality claims about women experiences. Hence this limitation calls out for the importance of conducting longitudinal studies on IPV and WASH. Despite utilising the modified DHS questionnaire that is mostly used to measure domestic violence from participants, the study did not include measures of controlling behaviours as a form of IPV.

## CONCLUSIONS

Intimate partner violence is a pervasive and serious public health problem affecting women in urban slums. Despite different factors known to be associated with IPV, this study was able to significantly link association of IPV and accessibility to WASH. Exciting findings from this study found women who had access to water from outside households were at risk factor of IPV compared to women who had access within households. Women who had access to water and sanitation facilities in the past 24 hours were negatively associated with IPV. This may suggest that it’s not only sufficient to provide external water sources but ensure the availability is within the households. This study recommends effective improvement of accessibility of water and sanitation facilities to further limit IPV among households in Kibra slums. Further research using longitudinal studies and qualitative methods may be explored to strengthen and unearth findings.

## Additional material


Online Supplementary Document
