# Supplementary material for: Water, sanitation, and intimate partner violence: Insights from Kibra Slums, Nairobi
Source: J Glob Health. 2024 Jun 28;14:04141. doi: 10.7189/jogh.14.04141 (PMC11211967; doi:10.7189/jogh.14.04141)
Supplement: Online Supplementary Document [file jogh-14-04141-s001.pdf]

Supplementary Material

Table 1: Sociodemographic characteristics	N=1068
**Variables**	**n (%)**
**Respondent's Age (Mean and SD)***	30.4(7.5)
15-24	263(24.6)
25-34	496(46.4)
35 and above	309(28.9)
**Partner or Ex-partner's Age (Mean and SD)***	35.3(8.8)
15-24	95(8.9)
25-34	435(40.7)
35 and above	538(50.4)
**Age Difference (Mean and SD)***	5.3(4.4)
0 to 2	261(24.4)
3 to 5	421(39.4)
6 to 10	304(28.5)
Above 10	82(7.7)
**Respondent's Education, level**	
Primary	431(40.4)
Secondary	507(47.5)
College/TVET	108(10.1)
University	22(2.1)
**Partner or Ex-partner's Education,level**	
Primary	301(28.2)
Secondary	554(51.9)
College/TVET	164(15.4)
University	49(4.6)
**Marital status**	
Single	733(68.6)
Married	114(10.7)
Divorced	85(8.0)
Cohabiting	123(11.5)
Widowed	13(1.2)
Years in Marriage (Mean and SD)*	22.3(12.0)
**Partner has another wife**	
Yes	178(16.8)
No	883(83.2)
**Number of Children**	
0 to 1	322(30.2)
2 to 4	639(59.8)

Table 1: Sociodemographic Characteristics

5 and above	107(10.0)
**Village**	
Makina	122(11.4)
Gatwekera	214(20.0)
Lindi	211(19.8)
Laini saba	135(12.6)
Kambi Muru	120(11.2)
Kianda	161(15.1)
Mashimoni	105(9.8)
**Respondent's employment type**	
Employed	126(11.8)
Self-employed	317(29.7)
Unemployed	625(58.5)
**Partner or Ex-partner employment type**	
Employed	513(48.0)
Self-employed	307(28.8)
Unemployed	248(23.2)
**Average household monthly income**	
15,000>	293(27.4)
<15,000	775(72.6)

Table 2: Prevalence of the different components of IPV

	**IPV N(1068)**	
	**Yes**	**No**
**Variable**	**n (%)**	**n (%)**
Experienced any IPV	684(64.0)	384(36)
Sexual violence	90(8.4)	978(91.6)
Emotional violence	385(36.1)	683(63.9)
Physical violence	528(49.4)	540(50.6)

Table 3: Environmental Determinants of IPV

**Variable**	**IPV**		**COR(95% CI)**	**P-value**
	**Yes**	**No**		
**Operated land for agriculture**				
No*	628(63.9)	355(36.1)	-	-
Yes	50(64.1)	28(35.9)	1.00(0.62-1.63)	0.969
**Raised any livestock**				
No*	616(64.3)	342(35,7)	-	-
Yes	62(60.2)	41(39.8)	0.84(0.55-1.27)	0.41
**Responsible for household water consumption**				
Wife*	155(63.0)	91(37.0)	-	-
Partner	523(64.2)	292(35.8)	0.95(0.71-1.28)	0.739
**Access to primary water**				
No*	383(71.6)	152(28.4)	-	-
Yes	301(56.5)	232(43.5)	**0.51(0.50-0.66)**	**<0.001**
**How primary water source is accessed**				
Inside household*	3(75.0)	1(25.0)	-	-
Outside tap/Public tap/Well	287(70.5)	120(29.5)	**2.75(1.68-4.50)**	**<0.001**
Vendor/tanker/burst pipe/Stream	394(60)	263(40.0)	**3.88(2.33-6.45)**	**<0.001**
**Groceries/Fish mongering Business**				
No*	608(63.4)	351(36.6)	-	-
Yes	70(68.6)	32(31.4)	1.26(0.81-1.96)	0.297
**Income impacted by adverse climatic conditions**				
No*	377(64.7)	206(35.3)	-	-
Yes	307(63.3)	178(36.7)	0.94(0.73-1.21)	0.643
**Decreased size of ordering groceries/fish**				
No*	22(71.0)	9(29.0)	-	-
Yes	48(67.6)	23(32.4)	0.85(0.34-2.14)	0.737
**Access to toilet**				
No*	255(72.0)	99(28.0)	-	-
Yes	429(60.1)	285(39.9)	**0.58(0.44-0.77)**	**<0.001**

Table 4. Socio-Behavioral Determinants of IPV

Variable	IPV		COR(95% CI)	P-value
	Yes n(%)	No n(%)		
**Exposure to IPV**				
No*	370(58.0)	268(42.0)	-	-
Yes	314(73.0)	116(27.0)	**1.96(1.50-2.55)**	**<0.001**
**Partner exposed to IPV**				
No*	504(60.9)	324(39.1)	-	-
Yes	180(75.0)	60(25.0)	**1.93(1.40-2.67)**	**<0.001**
**Childhood sexual abuse**				
No*	617(62.9)	364(37.1)	-	-
Yes	67(77.0)	20(23.0)	**1.98(1.18-3.31)**	**0.01**
**Partner's childhood physical abuse**				
Don't Know *	348(62.4)	210(37.6)	-	-
Yes	59(71.1)	24(28.9)	1.48(0.90-2.46)	0.125
No	277(64.9)	150(35.1)	1.11(0.86-1.45)	0.418
**IPV an acceptance norm**				
No*	580(62.7)	345(37.3)	-	-
Yes	104(72.7)	39(27.3)	**1.59(1.07-2.35)**	**0.021**
**Takes alcohol**				
No*	608(61.9)	375(38.1)	-	-
Yes	68(89.5)	8(10.5)	**5.24(2.49-11.03)**	**<0.001**
**Partner takes alcohol**				
No*	414(54.1)	351(45.9)	-	-
Yes	262(89.1)	32(10.9)	**6.94(4.68-10.29)**	**<0.001**
**Sought help**				
No*	530(58.8)	372(41.2)	-	-

Yes	154(92.8)	12(7.2)	**9.01(4.93-16.45)**	**<0.001**

Table 5. Multivariable Logistic Regression

**Multivariable binary logistic regression**	IPV			
	**Yes n(%)**	**No n(%)**		
**Variable**			**AOR(95% CI)**	**P-value**
**Respondent's Age**				
15-24*	149(56.7)	114(43.3)	-	-
25-34	327(65.9)	169(34.1)	1.27(0.76-2.12)	0.357
35 and above	208(67.3)	101(32.7)	0.78(0.38-1.58)	0.484
**Partner or Ex Age**				
15-24*	55(57.9)	40(42.1)	-	-
25-34	249(57.2)	186(42.8)	0.70(0.37-1.31)	0.263
35 and above	380(70.6)	158(29.4)	1.00(0.45-2.23)	0.995
**Age Difference**				
0 to 2*	153(58.6)	108(41.4)	-	-
3 to 5	257(61.0)	164(39.0)	1.04(0.70-1.55)	0.846
6 to 10	210(69.1)	94(30.9)	0.97(0.60-1.57)	0.909
Above 10	64(78.1)	18(21.9)	1.10(0.52-2.32)	0.805
**Respondent's Level of Education**				
College/TVET/University *	65(50)	65(50)	-	-
Primary	312(72.4)	119(27.6)	**1.95(1.05-3.62)**	**0.035**
Secondary	307(60.6)	200(39.4)	1.44(0.84-2.48)	0.189
**Partner or Ex Education, Level**				
College/TVET/University*	114(53.5)	99(46.5)	-	-
Primary	211(70.1)	90(29.9)	1.50(0.86-2.62)	0.151
Secondary	359(64.8)	195(35.2)	1.32(0.84-2.09)	0.233
**Village**				
Gatwekera*	148(69.2)	66(30.8)	-	-
Makina	77(63.1)	45(36.9)	0.94(0.52-1.70)	0.837
Lindi	111(52.6)	100(47.4)	**0.20(0.12-0.33)**	**<0.001**
Laini saba	75(55.6)	60(44.4)	**0.23(0.13-0.42)**	**<0.001**
Kambi Muru	110(91.7)	10(8.3)	**2.40(1.09-5.30)**	**0.03**
Kianda	117(72.7)	44(27.3)	1.06(0.62-1.83)	0.829
Mashimoni	46(43.8)	59(56.2)	**0.29(0.16-0.54)**	**<0.001**
**Partner has another wife**				

No*	140(68.7)	38(21.3)	-	-
Yes	538(60.9)	345(39.1)	1.46(0.89-2.41)	0.137
**Number of Children**				
0 to 1*	182(56.5)	140(43.5)	-	-
2 to 4	423(66.2)	216(33.8)	1.00(0.64-1.57)	0.999
5 and above	79(73.8)	28(26.2)	1.38(0.67-2.84)	0.376
**Access to primary water**				
No*	383(71.6)	152(28.4)	-	-
Yes	301(56.5)	232(43.5)	**0.44(0.31-0.64)**	**<0.001**
**How primary water source is accessed**				
Inside Household*	3(75.0)	1(25.0)	-	-
Outside tap/Public tap/Well	287(70.5)	120(29.5)	**18.18(8.62-38.33)**	**<0.001**
Vendor/tanker/burst pipe/Stream	394(60)	263(40.0)	**14.42(6.88-30.24)**	**<0.001**
**Access to toilet**				
No*	255(72.0)	99(28.0)	-	-
Yes	429(60.1)	285(39.9)	**0.57(0.37-0.88)**	**0.01**
**Exposure to IGT**				
No*	370(58.0)	268(42.0)	-	-
Yes	314(73.0)	116(27.0)	**2.27(1.56-3.29)**	**<0.001**
**Partner exposed to IGT**				
No*	504(60.9)	324(39.1)	-	-
Yes	180(75.0)	60(25.0)	**1.59(1.01-2.48)**	**0.043**
**Childhood sexual abuse**				
No*	617(62.9)	364(37.1)	-	-
Yes	67(77.0)	20(23.0)	0.88(0.45-1.72)	0.712
**IPV an acceptance norm**				
No*	580(62.7)	345(37.3)	-	-
Yes	104(72.7)	39(27.3)	1.03(0.62-1.71)	0.9
**Takes alcohol**				
No*	608(61.9)	375(38.1)	-	-
Yes	68(89.5)	8(10.5)	2.25(0.90-5.62)	0.081
**Partner takes alcohol**				
No*	414(54.1)	351(45.9)	-	-
Yes	262(89.1)	32(10.9)	**5.35(3.39-8.45)**	**<0.001**
**Sought help**				
No*	530(58.8)	372(41.2)	-	-
Yes	154(92.8)	12(7.2)	**10.49(5.36-20.53)**	**<0.001**
